# Early diagnosis and therapy of Chlamydia psittaci pneumonia: insights from chest CT, tNGS, and omadacycline in 34 cases

**DOI:** 10.3389/fmed.2026.1921406

**Published:** 2026-08-28

**Authors:** Qiuting Jiang, Xiaoli Zhao, Yuan Zhu, Xinmin Zhu, Shaohua Hu, Huajuan Zhou

**Affiliations:** Department of Respiratory and Critical Care Medicine, Hangzhou Linping Hospital of Interated Traditional Chinese and Western Medicine, Zhejiang, China

**Keywords:** chest CT, Chlamydia psittaci pneumonia, early diagnosis, omadacycline, t targeted next-generation sequencing (tNGS)

## Abstract

**Objective:**

This study retrospectively analyzed the clinical characteristics and management strategies of 34 patients with *Chlamydia psittaci* pneumonia. We aimed to evaluate the diagnostic value of subpleural-predominant pulmonary CT manifestations combined with bronchoalveolar lavage fluid targeted next-generation sequencing (tNGS), and to assess the clinical efficacy and therapeutic advantages of omadacycline in this patient cohort.

**Methods:**

Clinical data were retrospectively collected from patients diagnosed with *C. psittaci* pneumonia by tNGS/PCR, from January 2024 to April 2026. Demographic characteristics, epidemiological history, clinical manifestations, laboratory findings, imaging features, treatment regimens, and clinical outcomes were analyzed.

**Results:**

A total of 34 patients were enrolled. Twenty-four patients (70.6%) had a history of close contact with birds or avian excreta. All patients developed fever, with a mean maximum body temperature of 39.6 ± 0.5°C, and fever was the predominant initial symptom in 29 patients (85.3%). Laboratory findings were characterized by elevated C-reactive protein levels in all patients. Chest imaging predominantly showed subpleural lesions presenting as consolidation, and the typical reverse halo sign was observed in 5 patients (14.7%). 30 patients diagnosed by tNGS and 4 diagnosed by PCR. The median *C. psittaci* sequence count detected by tNGS in bronchoalveolar lavage fluid (BALF) was 3,773.5 reads (IQR,588.5–11,856.0). Compared with non-omadacycline regimens, omadacycline was associated with a shorter time to defervescence (1.0 vs. 3.0 days, *p* < 0.001) and a greater reduction in C-reactive protein levels after 5 days of treatment (143.6 ± 63.2 mg/L vs. 87.4 ± 57.9 mg/L, *p* < 0.05), Notably, omadacycline demonstrated considerable clinical efficacy even in patients who had failed initial quinolone therapy. All patients improved and were discharged after effective antimicrobial therapy.

**Conclusion:**

*C. psittaci* pneumonia may occur without a clear epidemiological history and typically presents with high fever and nonspecific laboratory abnormalities. Subpleural consolidation and the reverse halo sign on chest imaging may provide important diagnostic clues. BALF tNGS is valuable for the early identification of *C. psittaci* infection, and omadacycline may be an effective therapeutic option, particularly in patients with persistent fever after quinolone therapy.

## 1 Introduction

Human psittacosis is a zoonotic infectious disease which is caused by the obligate intracellular bacterium *Chlamydophila psittaci* (*C. psittaci)*, primarily transmitted through inhalation of aerosols from contaminated urine, respiratory and ocular secretions, or contact with dried feces or feathers from infected animals or asymptomatic carriers (1). In a meta-analysis of 57 studies, *C. psittaci* was identified as the causative pathogen in 1.03% of tested community-acquired pneumonia cases (95% CI: 0.79–1.30) (2). *C. psittaci* pneumonia usually presents with nonspecific clinical symptoms, including fever, cough, dizziness, nausea, abdominal pain, poor appetite, and fatigue. However, delayed recognition may lead to severe pneumonia, multiorgan failure, or even death (3).Therefore, timely diagnosis and appropriate antimicrobial therapy is essential.

However, the nonspecific nature of early clinical manifestations and radiological features poses a significant challenge to the timely diagnosis of *Chlamydia psittaci* pneumonia (4).In recent years, targeted next-generation sequencing (tNGS), which combines multiplex polymerase chain reaction amplification with high-throughput sequencing, has emerged as a rapid and sensitive approach for detecting respiratory pathogens. Accumulating evidence indicates that targeted next-generation sequencing (tNGS) offers diagnostic performance comparable to metagenomic NGS (mNGS) for identifying respiratory pathogens (5).Regarding antimicrobial therapy, Although emerging case reports have documented favorable outcomes with omadacycline in severe *Chlamydia psittaci* pneumonia, high-level evidence—particularly from comparative cohort studies—remains lacking (6).

Building on this context, we conducted a retrospective analysis of a 34-patient cohort with *Chlamydia psittaci* pneumonia. This study aimed to characterize the clinical profiles, treatment responses, and prognostic outcomes, evaluate the clinical utility of subpleural involvement on CT and tNGS of bronchoalveolar lavage fluid for early diagnosis, and further delineate the distinct therapeutic advantages of omadacycline over other antibiotics.

## 2 Materials and methods

### 2.1 Study participants

This single-center retrospective cohort study retrospectively included patients diagnosed with *Chlamydia psittaci* pneumonia by tNGS/PCR hospitalized at Linping District Hospital of Integrated Traditional Chinese and Western Medicine in Hangzhou, Zhejiang Province, from January 2024 to April 2026. The inclusion criteria were: (1) diagnosed with community-acquired pneumonia (CAP) according to the current guideline (7); (2) positive tNGS/qPCR results of *C. psittaci* in bronchoalveolar lavage fluid (BALF). This study has been approved by the Ethics Committee of Linping District Integrated Traditional Chinese and Western Medicine Hospital in Hangzhou, Zhejiang Province (Approval Number: 2026-L-016).

### 2.2 Data collection

Demographic information, clinical data, laboratory findings, chest computed tomography (CT) features, bronchoscopy findings, and tNGS results from BALF were retrieved from the electronic medical system. Information on antimicrobial treatment and clinical outcomes was also documented.

### 2.3 Targeted next-generation sequencing (tNGS)

Bronchoalveolar lavage fluid (BALF) samples were sent to a certified clinical laboratory (Adicon Clinical Laboratories, Hangzhou, China) for tNGS testing. Prior to nucleic acid extraction, dedicated pre-lysis processing of BALF specimens was performed: samples were aliquoted and subjected to liquefaction, enzymatic digestion, and mechanical disruption via bead-beating to ensure efficient breakage of thick-walled or obligate intracellular organisms (including *Chlamydia* spp.). Subsequently, multiplex PCR amplification targeting clinically relevant pathogens was carried out, followed by library construction and high-throughput sequencing for pathogen identification and profiling within the assay's detection panel.

### 2.4 Statistical analysis

Statistical analyses were performed using SPSS version 27.0 (IBM Corp., Armonk, NY, USA). For categorical variables, data were presented as frequencies and percentages, and compared using Fisher's exact tests due to the small number less than 40 of our study. Normally distributed continuous variables were presented as mean ± standard deviation, whereas non-normally distributed variables were reported as median (interquartile range). For comparisons between two groups, normally distributed continuous variables were analyzed using Student's *t*-test, while non-normally distributed continuous variables were analyzed using the Mann–Whitney *U*-test. A *p* < 0.05 was considered statistically significant.

### 2.5 Quality control standards

All objective data from laboratory tests and examinations were obtained from hospitalized patients at Linping District Hospital of Integrated Traditional Chinese and Western Medicine in Hangzhou, Zhejiang Province. Laboratory results were achieved from the Department of Laboratory Medicine, while imaging data were obtained from the Department of Radiology; all data are authentic and comparable. The interpretation of imaging findings was given by two respiratory physicians with clinical expertise and one radiologist.

## 3 Result

### 3.1 Demographic characteristics

This study included a total of 34 patients with *C. psittaci* pneumonia, comprising 17 males (50.0%) and 17 females, with an average age of 59.8 ± 13.4 years. Among the patients, 16 (47.1%) had underlying medical conditions, including hypertension in 8 patients (23.5%), diabetes mellitus in 5 (14.7%), colorectal malignancy in 2 (5.9%), chronic obstructive pulmonary disease in 2 (5.9%), bronchiectasis in 1 (2.9%), rheumatoid arthritis in 1 (2.9%), hypothyroidism in 1 (2.9%), chronic hepatitis B surface antigen carrier status in 1 (2.9%), and herpes zoster infection in 1 (2.9%).

24 patients (70.6%) reported close contact with poultry or related excreta, of whom 11 (32.3%) had contact with parrots, 6 (17.6%) with chickens or ducks, 4 (11.8%) visited live poultry markets, 2 (5.9%) had contact with discarded bird cages, and 1 (17.6%) had contact with pigeons. The remaining 10 patients denied a clear history of poultry or bird exposure. Notably, two clustered outbreaks were identified in this cohort, involving four and two confirmed patients, respectively. In both clusters, the affected patients had shared exposure to sick parrots, suggesting avian-to-human transmission, with no clear evidence supporting human-to-human transmission.

The majority of cases (27, 79.41%) occurred between October and February, including 10 cases (29.4%) in January. In December, eight cases were reported (23.5%); see Table 1 for details.

**Table 1:** Time and results of tNGS in alveolar lavage fluid from 30 patients with *C. psittaci* pneumonia.

Case	Number of sequences for Chlamydia psittaci	Time from onset of disease to collection of bronchoscopic alveolar lavage fluid for tNGS analysis (days)	Other pathogens in tNGS
3	6,656	6	*Klebsiella pneumoniae*
5	256	15	/
6	2,357	7	/
8	43,922	9	/
9	22,989	10	/
10	754	6	EB virus
11	5,025	7	coronavirus
12	4,741	7	Human herpesvirus type 1, rhinovirus
13	1,56,856	9	/
14	5,469	6	*Staphylococcus aureus*
15	421	4	*Haemophilus parainfluenzae*
16	626	10	*Aspergillus niger*
17	472	4	*Staphylococcus aureus, Pneumocystis jirovecii*, EB virus
18	476	9	/
19	329	7	/
20	10,376	10	*Aspergillus fumigatus*
21	1,672	7	*Tropheryma whipplei*
22	4,874	10	*Staphylococcus aureus, Klebsiella pneumoniae*
23	2,756	7	Rhinovirus
24	18,310	8	*Aspergillus flavus, Aspergillus niger*, human herpesvirus
25	16,296	5	/
26	120	6	/
27	2,886	5	*Staphylococcus aureus*
28	4,661	8	/
29	1,099	6	/
30	5,772	9	/
31	20,622	5	/
32	199	6	*Cryptococcus neoformans*
33	670	4	/
34	16,478	15	/

### 3.2 Clinical manifestations

Fever was the initial symptom in 29 patients (85.3%), followed by cough in 5 patients (14.7%). During the disease course, the most common accompanying symptoms were fever (34/34, 100%), cough (32/34, 94.1%), fatigue (31/34, 91.2%), poor appetite (28/34, 82.4%), myalgia (21/34, 61.8%), and headache (21/34, 61.8%). Less frequent symptoms included chest tightness (10/34, 29.4%), apathy (4/34, 11.8%), chest pain (3/34, 8.8%), diarrhea (2/34, 5.9%), and abdominal pain (1/34, 2.9%). Respiratory failure occurred in 4 patients (11.8%), all of whom required high-flow nasal cannula oxygen therapy. Details are presented in Supplementary Table S1.

### 3.3 Laboratory findings

Leukocytosis was observed in 8 patients (23.5%), with a median white blood cell count of 7.3 × 10^9^/L (IQR,6.0–9.5 × 10^9^/L). Neutrophilia was more common, occurring in 32 patients (94.1%), and the median neutrophil percentage was 82.7% (IQR,75.3–85.4%). C-reactive protein was elevated in all patients, with a mean level of 143.1 ± 72.9 mg/L, whereas procalcitonin was elevated in 10 patients (31.3%), with a median level of 0.3 ng/mL IQR, 0.1–0.8 ng/mL). Evidence of liver function abnormalities was also observed. Aspartate aminotransferase was elevated in 18 patients (52.9%), with a median level of 51.0 U/L (IQR, 24.0–78.5 U/L), and alanine aminotransferase was elevated in 16 patients (47.1%), with a median level of 44.5 U/L (IQR, 24.3–85.3 U/L). Gamma-glutamyl transferase elevation was present in 15 patients (44.1%), with a median level of 33.0 U/L (IQR, 16.5–96.3 U/L). Other abnormalities included elevated lactate dehydrogenase in 15 patients (45.5%), with a mean level of 256.6 ± 77.1 U/L, and elevated creatine kinase in 12 patients (36.4%), with a median level of 118.0 U/L (IQR, 62.5–280.0 U/L). Hypokalemia was found in 6 patients (18.2%), with a mean potassium level of 3.9 ± 0.5 mmol/L. Decreased creatinine clearance was observed in 10 patients (30.3%), with a mean value of 92.3 ± 28.0 mL/min. D-dimer elevation was noted in 23 patients (69.7%), with a median level of 879.0 ng/mL (IQR, 516.5–1,707.0 ng/mL). Details are presented in Supplementary Table S2.

### 3.4 Imaging findings

**Figure 1:**
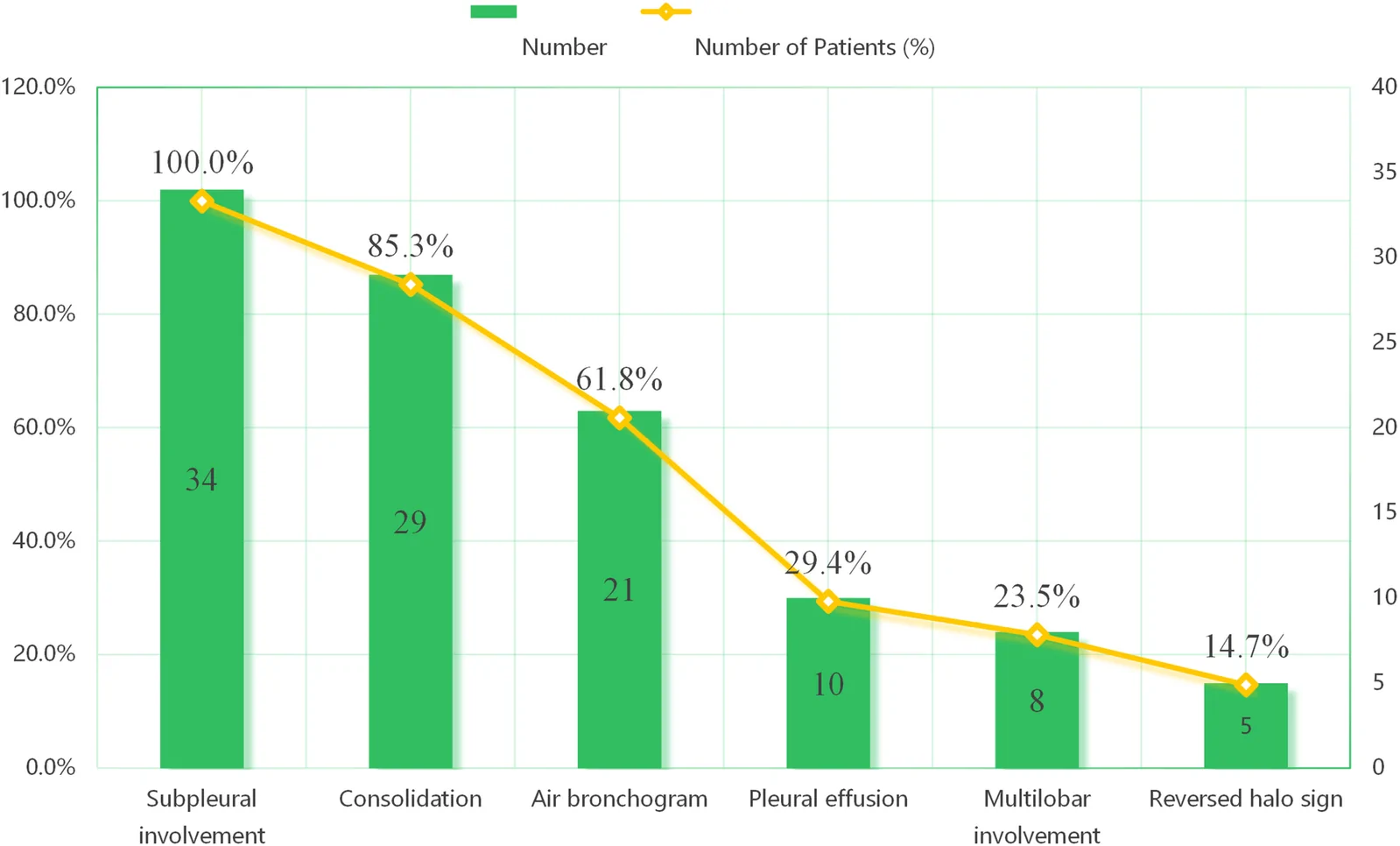
Chest CT characteristics of 34 patients with *C. psittaci* pneumonia.

**Figure 2:**
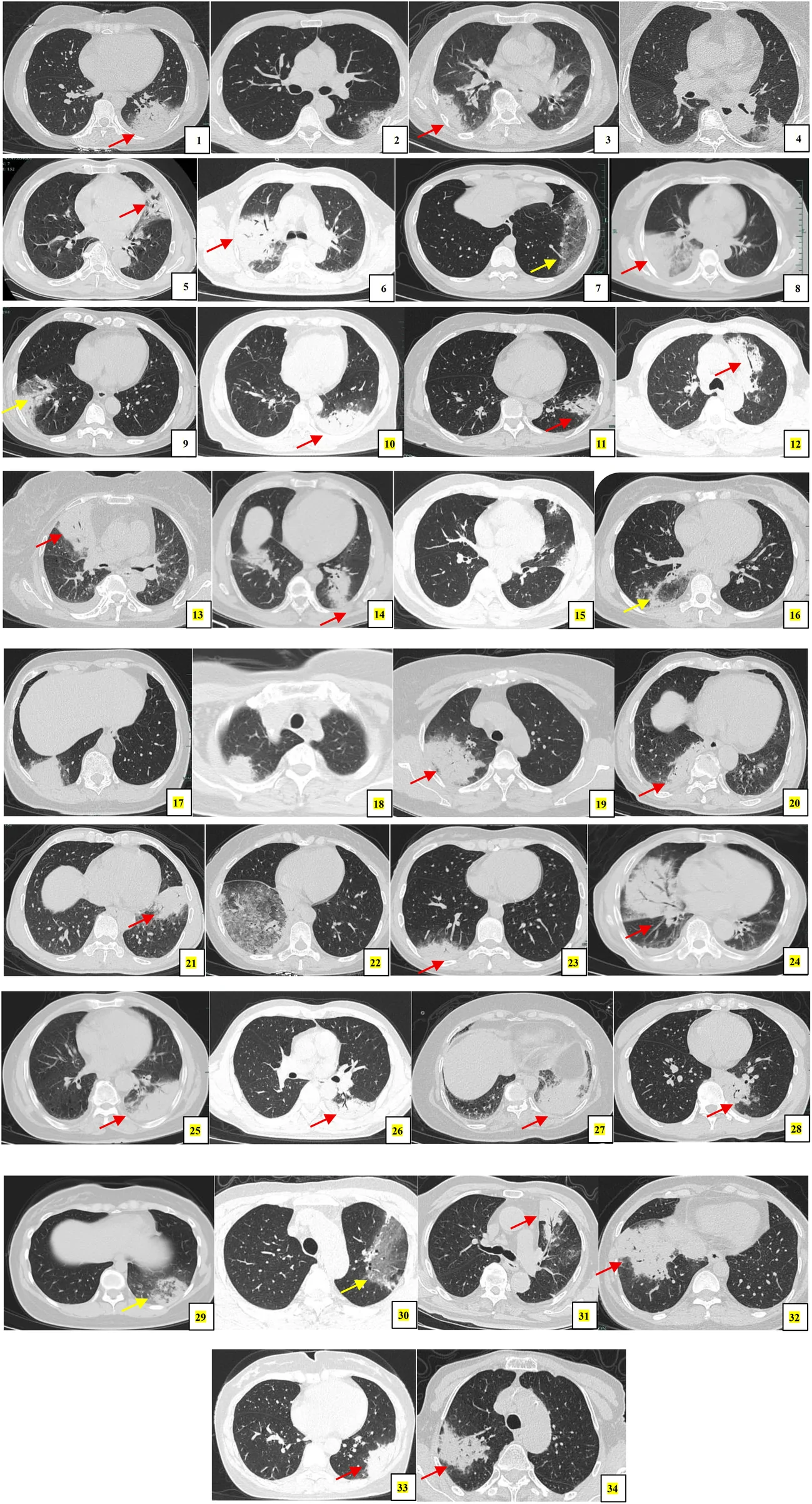
Chest CT scans of 34 patients with *C. psittaci* pneumonia.

Chest imaging predominantly showed lobar pneumonia, and all patients had subpleural involvement. Involvement of two or more lung lobes was observed in 8 patients (23.5%). Consolidation was observed in 29 patients (85.3%), among whom 21 (61.8%) showed air bronchograms. Pleural effusion was present in 10 patients (29.4%), and the typical halo sign was identified in 5 patients (14.7%). Representative imaging findings are shown in Figures 1, 2.

Case 1:Consolidation in the left lower lobe with ill-defined margins, air bronchograms, and a minimal left pleural effusion. Case 2: Patchy consolidation in the left lower lobe with ill-defined margins. Case 3:Patchy consolidation in the left lingular segment and right lower lobe with ill-defined margins. Air bronchograms were noted, predominantly in the right lung. Case 4:Patchy consolidation in the right upper lobe and bilateral lower lobes with ill-defined margins. Case 5:Consolidation in the left lingular segment with ill-defined margins, air bronchograms, and left pleural effusion. Case 6: Consolidation in the right upper and middle lobes with air bronchograms, surrounded by patchy ground-glass opacities, and minimal bilateral pleural effusions. Case 7: Ground-glass opacity in the right lower lobe with a reversed halo sign. Case 8: Consolidation in the right lower lobe with air bronchograms and ill-defined margins, accompanied by right pleural effusion. Case 9:Patchy consolidation in the right lower lobe with ill-defined margins and a reversed halo sign. Case 10:Consolidation in the left lower lobe with air bronchograms. Case 11: Irregular patchy consolidation in the left upper and lower lobes with ill-defined margins and air bronchograms. Case 12:Multiple patchy consolidations in the left upper lobe with ill-defined margins and air bronchograms. Case 13:Consolidation in the right middle lobe with air bronchograms and right pleural effusion. Case 14:Consolidation in both lower lobes with air bronchograms. Case 15:Patchy consolidation in the left upper lobe with ill-defined margins. Case 16:Patchy consolidation in the right lower lobe with ill-defined margins and a reversed halo sign. Case 17:Consolidation in the right lower lobe with ill-defined margins. Case 18:Mass-like consolidation in the right upper lobe with ill-defined margins. Case 19:Patchy consolidation in the right upper lobe with ill-defined margins and air bronchograms. Case 20: Consolidation in the right lower lobe with air bronchograms and ill-defined margins. Case 21: Consolidation in the left lower lobe with air bronchograms. Case 22:Patchy consolidation in the right lower lobe with partial consolidation and ill-defined margins. Case 23:Consolidation in the right lower lobe with surrounding exudation, pleural thickening, and air bronchograms. Case 24:Consolidation in the right middle lobe with air bronchograms and bilateral pleural effusions. Case 25:Consolidation in the left lower lobe with air bronchograms and ill-defined margins. Case 26: Consolidation in the left lower lobe with air bronchograms and ill-defined margins. Case 27:Consolidation in the left lingular segment and lower lobe with air bronchograms and ill-defined margins, accompanied by left pleural effusion. Case 28:Patchy consolidation in the left lower lobe with air bronchograms and ill-defined margins. Case 29:Patchy consolidation in the left lower lobe with a reversed halo sign. Case 30:Patchy consolidation in the right upper lobe with ill-defined margins and a reversed halo sign. Case 31: Consolidation in the left upper lobe with air bronchograms and ill-defined margins. Case 32:Consolidation in the right lower lobe with air bronchograms and ill-defined margins. Case 33: Patchy consolidation in the right middle lobe and bilateral lower lobes with air bronchograms and ill-defined margins. Case 34:Consolidation in the right upper lobe with air bronchograms and ill-defined margins.

### 3.5 Microorganism detection results

Bronchoscopy was performed in all patients. During the procedure, the bronchoscope was wedged into the affected subsegmental bronchus, and BALF was obtained by instilling and aspirating sterile saline in three 20 mL aliquots. Bronchoscopy was performed in all patients to obtain BALF. Whereas 30 patients were diagnosed via tNGS, 4 patients with a clear history of avian exposure and familial clustering among confirmed cases bypassed tNGS and proceeded directly to *C. psittaci* PCR testing. All collected BALF samples were subsequently forwarded to the Linping District Center for Disease Control and Prevention in Hangzhou, Zhejiang Province, for confirmatory qPCR, which validated all initial findings. The median *C. psittaci* sequence count of BALF tNGS was 3,773.5 reads (IQR, 588.5–11,856.0). The median interval from symptom onset to the availability of tNGS results was 7.0 days (IQR, 6.0–9.0 days). Co-detected pathogens included *Staphylococcus aureus* in 4 patients (13.3%) and *Aspergillus spp.* in 3 patients (10.0%). Details are shown in Table 1.

### 3.6 Correlation between bronchoalveolar lavage fluid (BALF) TNGS results and disease severity

We further evaluated the correlations between bronchoalveolar lavage fluid (BALF) TNGS sequencing counts and clinical parameters, including the CURB-65 score, radiological findings (consolidation and multilobar infiltration), and systemic inflammatory biomarkers (CRP and PCT). Spearman rank correlation analysis revealed significant positive correlations between TNGS read counts and both CRP (*ρ* = 0.447, *p* = 0.013) and PCT (*ρ* = 0.514, *p* = 0.004), whereas no significant correlation was observed with the CURB-65 score (*ρ* = 0.239, *p* = 0.204). Furthermore, Mann–Whitney *U*-tests showed no statistically significant differences in TNGS levels between patients with vs. without pulmonary consolidation (*Z* = −0.449, *p* = 0.653) or between those with vs. without multilobar infiltrates (*Z* = −0.368, *p* = 0.713).

### 3.7 Treatments response and outcomes

Before receiving effective anti-*C. psittaci* therapy, such as tetracyclines or quinolones, all patients had persistent clinical symptoms, predominantly fever, and many showed progressive worsening. Symptoms resolved after the diagnosis was confirmed and the antimicrobial therapy was modified accordingly. The median interval from symptom onset to hospitalization was 4.0 days (IQR, 3.0–5.3 days), and the median length of hospital stay was 9.0 days (IQR, 7.0–11.0 days). Details are presented in Supplementary Table S3.

Patients were divided into two groups according to the post-diagnosis antimicrobial regimen. Group A received omadacycline(loading dose 200 mg, followed by 100 mg once daily, administered intravenously), whereas Group B received non- omadacycline regimens, including quinolones alone, quinolones combined with doxycycline, ciprofloxacin, and azithromycin. Compared with Group B, Group A had a shorter time to defervescence (1.0 vs. 3.0 days, *p* < 0.001) and a greater reduction in C-reactive protein levels 5 days after treatment (143.6 ± 63.2 mg/L vs. 87.4 ± 57.9 mg/L, *p* < 0.05; Table 2). Notably, among patients initially treated with quinolones, 15 experienced persistent fever. Following a switch to omadacycline, these patients exhibited marked clinical improvement, with defervescence achieved after a mean of 1.1 ± 0.4 days. All patients exhibited good tolerability, and no notable adverse reactions occurred throughout the treatment course.

**Table 2:** Comparison of treatment regimens for 34 patients with *C. psittaci* pneumonia.

Divide into groups	Total (*N* = 34)	Group A (*N* = 21)	Group B (*N* = 13)	*p*
Time to decrease body temperature	2.0 (1.0,2.3)	1.0 (1.0,2.0）	3.0 (2.0,3.5)	<0.001
CRP level (mg/L) at disease onset	143.1 + 72.9	168.2 + 69.3	102.7 + 61.24	0.009
After 5 days: CRP level (mg/L)	14.9 (8.5,23.2)	16.7 (10.1,23.6)	7.4 (5.2,23.6)	0.038
CRP levels decrease after 5 days (mg/L)	122.1 + 66.4	143.6 + 63.2	87.4 + 57.9	0.014

## 4 Discussion

Our study enrolled 34 patients diagnosed with *C. psittaci* pneumonia. 30 patients were diagnosed by tNGS and 4 were diagnosed by PCR. The most common route of exposure to *C. psittaci* is inhalation of contaminated material, such as dried feces or other secretions. Besides parrots, poultry—including pigeons, chickens, turkeys, geese, and ducks—also serve as important sources of infection (3). In the present study, 24 patients (70.5%) had a history of close contact with birds or avian excreta, which is consistent with other retrospective studies in which only 23.2%–27% of patients denied any history of avian contact (8, 9). Among these patients, two had visited live-poultry markets without direct contact with the birds, and two reported contact with discarded birdcages, suggesting possible fecal transmission. Two clusters of cases warrant particular attention: The first involved a family of four who kept a pet parrot on their balcony: two members had direct contact with the bird, whereas the other two did not. Because existing epidemiological evidence suggests human-to-human transmission of *C. psittaci* (10), this potential mode of transmission should not be overlooked. The second cluster comprised a mother and daughter, both of whom had handled or fed a diseased parrot.

Regarding demographic characteristics, in our study, the mean age of the patients was 59.8 ± 13.4 years, and 16 patients (47.1%) had underlying comorbidities. This finding is consistent with previous reports indicating that *C. psittaci* infections can occur in both immunocompetent individuals and patients with underlying diseases (11).

Psittacosis is a zoonotic disease that can involve the respiratory tract, heart, kidneys, nervous system, hematologic system, and osteoarticular system. Clinical manifestations of *C. psittaci* pneumonia mainly include fever, headache, sore throat, myalgia, cough and expectoration, anorexia, diarrhea, altered mental status, arthralgia, chest tightness, and chest pain (12), Rare cases have reported cerebellar dysfunction (13), epilepsy (14), and rhabdomyolysis (15). In this study, most patients presented with high fever (85.3%), followed by cough, fatigue, poor appetite, headache, chest tightness, apathy, abdominal pain, diarrhea, hemoptysis, and chest pain. Notably, two patients (5.9%) did not develop respiratory symptoms such as cough, chest tightness, or chest pain throughout the disease course, and four patients (11.7%) presented with apathy. The atypical respiratory symptoms posed challenges for the clinical diagnosis and treatment of the disease.

In terms of laboratory examination, the findings in Chlamydia psittaci pneumonia were nonspecific. White blood cell count, neutrophil percentage, lactate dehydrogenase, alanine aminotransferase, aspartate aminotransferase, *γ*-glutamyl transferase, creatine kinase, and procalcitonin could be normal or mildly elevated; creatinine clearance and serum potassium could be normal or mildly decreased; and high-sensitivity C-reactive protein was elevated in all cases, broadly consistent with the laboratory findings in previous studies (12).

Regarding imaging findings, existing literature suggests that lesions may involve a single lobe or multiple lobes. Common imaging features include consolidation, interlobular septal thickening, air bronchograms, and the reverse halo sign. Some patients may present with pleural effusion or mediastinal lymphadenopathy, whereas tree-in-bud signs, necrosis, or cavitation are generally absent (16, 17). In this study, the reversed halo sign was observed on CT in five patients (14.7%), with CT performed 7.0 (6.0, 7.0) days after onset. Previous studies have proposed that the reverse halo sign in *C. psittaci* pneumonia may be associated with lesion absorption beginning in the central vascularized area, which is consistent with the relatively late timing of CT examination in this study (17). Because this sign is rarely observed in other pulmonary diseases, it remains of important diagnostic value. In addition, all patients in the present study showed subpleural involvement on imaging. Similar CT findings have also been observed in other reported cases of psittacosis (18, 19), suggesting that subpleural involvement may also represent one of the characteristic imaging features of this disease.

Traditional methods for detecting Chlamydia psittaci infection primarily include pathogen culture, serological testing, or polymerase chain reaction (PCR) (20). However, culture for *C. psittaci* is time-consuming and requires high laboratory biosafety standards, making it unsuitable for routine use. Serological testing has limited value for early diagnosis. Although quantitative polymerase chain reaction (qPCR) monitoring for *C. psittaci* is considered the diagnostic gold standard, it is difficult to obtain in routine clinical practice (21). Metagenomic next-generation sequencing (mNGS) is effective and accurate in identification of pathogenic microorganisms, and plenty of studies have relied on mNGS for the diagnosis of *C. psittaci* pneumonia (22). Nevertheless, this method has drawbacks such as high cost, long turnaround time, and contamination of host-derived genetic materials, limiting its clinical application. TNGS is a novel detection method that captures and sequences pathogen DNA and RNA in clinical samples and performs high-throughput sequencing of targeted regions. It is efficient and relatively inexpensive, and its high degree of automation substantially reduces the possibility of human-derived contamination (23). Several studies have demonstrated comparable sensitivity and specificity between tNGS and mNGS using bronchoalveolar lavage fluid samples have similar sensitivity and specificity in patients with pneumonia (24). Additionally, tNGS demonstrates high detection efficiency for atypical pathogenic microorganisms in the respiratory tract (25). In this study, all detections of *C. psittaci* by tNGS were consistent with PCR testing. Moreover, we noted that even when patients had initially received antibiotics with activity against *C. psittaci*, tNGS could still detect *C. psittaci*, which confirmed the important diagnostic value of tNGS in *C. psittaci* pneumonia. Notably, although tNGS is a valuable diagnostic modality, it shares inherent limitations with metagenomic NGS (mNGS). As a DNA-based assay, it cannot distinguish viable, replicating bacteria from residual DNA of lysed or dead organisms (26), and it carries a recognized risk of reagent-background or environmental microbiome contamination (27). Therefore, tNGS results must be interpreted with caution and never in isolation from the clinical context.

According to contemporary tNGS interpretation frameworks (28), analytical positivity for bacterial targets is defined as amplicon coverage ≥50% combined with a normalized read count (RPM) ≥10. However, analytical positivity alone is insufficient to establish causative pathogenicity; clinician adjudication is required, integrating culture data, radiological findings, inflammatory biomarkers, and host immune status to triage detected organisms into suspected pathogen, colonizer, or contaminant. Applying this framework to the four patients with BALF tNGS co-detection of *Chlamydia psittaci* and *Staphylococcus aureus*, *S.aureus* was uniformly classified as representing colonization, upper-airway carriage, or reagent-background contamination rather than active co-infection. This adjudication was substantiated by three key observations: (i) *S. aureus* exhibited quantitatively subordinate sequencing depth, with a median read count of 807 (range 199–1,353), markedly lower than that of co-detected *C. psittaci* (median 3,880; range 472–5,469); (ii) serial sputum cultures obtained on admission and the subsequent day were persistently negative across all four patients, indicating a low staphylococcal burden without active proliferation; (iii) chest CT scans lacked the canonical radiological stigmata of staphylococcal pneumonia—specifically necrotizing consolidation, thick-walled cavitation, or thin-walled pneumatoceles—findings well-established as hallmark CT signatures of *S.aureus* pulmonary infection (29).Collectively, these data affirm *C. psittaci* as the sole primary pathogen driving this clinical syndrome.

Before diagnosis of *C. psittaci* infection, all patients received empirical antibiotic therapy in accordance with community-acquired pneumonia guidelines; however, their clinical conditions showed no improvement or even progressively worsened. After diagnosed with *C. psittaci* infection and appropriate treatment was administered, all patients showed clinical improvement and were discharged successfully. Regarding the choice of antibiotics, tetracyclines, macrolides, and quinolones are currently available as anti-infective options for chlamydial infections (30), whereas tetracyclines are the first choice for *C. psittaci*, with quinolones as a second-line option (31). In this study, the omadacycline group had a shorter time to defervescence than the group receiving other antibiotics (1.0 day vs. 3.0 days, *p* < 0.05) and showed a more pronounced decrease in CRP on re-examination after 5 days (143.6 ± 63.2 mg/L vs. 87.4 ± 57.9 mg/L, *p* < 0.05); meanwhile, fever recurred in 15 patients during quinolone therapy but resolved after switch to omadacycline, yielding a favorable outcome in all, with a mean time to to defervescence of 1.1 ± 0.4 days. The above evidence supports omadacycline as a preferred treatment option for *C. psittaci* pneumonia that offers better efficacy and fewer adverse effects, consistent with results from other prospective studies (32).

This study has several limitations. First, as a single-center retrospective study with small samples, it may have been affected by confounding bias. Second, the follow-up period was limited to 1 month after discharge, and potential long-term complications were not systematically evaluated. Third, the sample size was relatively small, which may affect the reliability and generalizability of the findings. The therapeutic success of omadacycline in this context can be attributed to its pharmacological advantages over fluoroquinolones against *C. psittaci*. As an obligate intracellular pathogen, *C. psittaci* is poorly targeted by quinolones due to their limited intracellular bioavailability, corroborated by multiple reports of Fluoroquinolones failure (33). Omadacycline, a C7/C9-modified aminomethylcycline, evades the two principal tetracycline-resistance mechanisms (efflux pumps and ribosomal protection proteins) and achieves higher alveolar-cell concentrations than tigecycline or eravacycline (34, 35).Therefore, the marked clinical improvement upon switching to omadacycline after tNGS diagnosis likely reflects its enhanced intracellular activity and resistance profile. Nevertheless, the retrospective nature of this study limits causal inference. Future investigations featuring larger, multicenter cohorts and prospective interventional designs are warranted to validate these mechanistic hypotheses and further establish the role of omadacycline in the management of severe psittacosis.

## 5 Conclusion

Clinically, *C. psittaci* pneumonia often presents with high fever at onset. Laboratory findings are commonly characterized by elevated C-reactive protein levels. On imaging, the reverse halo sign, consolidation, and subpleural involvement may represent relatively specific features. Bronchoalveolar lavage fluid tNGS can improve the diagnostic rate of C. psittaci infection. In terms of treatment, both tetracyclines and quinolones are effective; however, compared with quinolone antibiotics, omadacycline has a more rapid onset of action and may serve as salvage therapy after quinolone treatment failure.

## Data Availability

The raw data supporting the conclusions of this article will be made available by the authors, without undue reservation.
