# Supplementary material for: Early diagnosis and therapy of Chlamydia psittaci pneumonia: insights from chest CT, tNGS, and omadacycline in 34 cases
Source: Front Med (Lausanne). 2026 Aug 28;13:1921406. doi: 10.3389/fmed.2026.1921406 (PMC13562005; doi:10.3389/fmed.2026.1921406)
Supplement: Supplementary file 1 [file Table1.docx]

Supplementary table 1 General demographic data of 34 patients with *C. psittaci* pneumonia

| **Case** | **Sex** | **Age** | **Occupation** | **Medical history** | **Contact history** | **Time from contact to onset of symptoms**  **（ days ）** | **Season of onset** | **Body temperature peak**  **（℃）** | **Initial symptoms** | **Simultaneous phenomenon** | **Oxygen therapy methods** |
| --- | --- | --- | --- | --- | --- | --- | --- | --- | --- | --- | --- |
| 1 | Woman | 47 | Office worker | Hypertension | Parrot | 7 | 2 | 39.4 | Cough | Nausea, fatigue, headache, muscle soreness | Standard oxygen therapy |
| 2 | Man | 55 | Civil servant | None | Parrot | 7 | 2 | 39 | Fever | Nausea, fatigue, muscle soreness | Standard oxygen therapy |
| 3 | Man | 75 | Retire | Hypertension, diabetes | Parrot | 6 | 2 | 39.2 | Cough | Nausea, fatigue | Standard oxygen therapy |
| 4 | Woman | 66 | Retire | Hypertension, rheumatoid arthritis | Parrot | 3 | 2 | 39.8 | Fever | Nausea, cough, fatigue, headache | Treatment with high-flow nasal ventilation |
| 5 | Man | 71 | Retire | Malignant tumor of colon | Chicken | 2 | 10 | 38.8 | Cough | Nausea, fatigue, abdominal pain, diarrhea, muscle soreness, headache | Standard oxygen therapy |
| 6 | Man | 77 | Retire | Hypertension | Has visited a live poultry market | 4 | 10 | 39.4 | Cough | Nausea, cough, fatigue, muscle soreness, headache | Treatment with high-flow nasal ventilation |
| 7 | Woman | 37 | Office worker | None | Parrot | 1 | 12 | 39 | Fever | Nausea, fatigue, muscle soreness | Standard oxygen therapy |
| 8 | Woman | 49 | Collect scrap | None | Birdcage | 5 | 1 | 40.6 | Fever | Nausea, cough, fatigue, muscle soreness, headache | Standard oxygen therapy |
| 9 | Man | 66 | Retire | None | Ornithocopros | 3 | 1 | 40 | Fever | Nausea, cough, fatigue, chest tightness, apathy | Standard oxygen therapy |
| 10 | Woman | 60 | Peasant | None | Live duck | 10 | 1 | 39 | Fever | Cough, fatigue, abdominal pain, myalgia, hemoptysis | Standard oxygen therapy |
| 11 | Woman | 57 | Office worker | Hypothyroidism | Chicken | 1 | 5 | 39.8 | Fever | Fatigue, muscle soreness, apathy | Standard oxygen therapy |
| 12 | Man | 66 | Worker | None | None | / | 3 | 39.6 | Fever | Cough, fatigue, muscle soreness | Standard oxygen therapy |
| 13 | Woman | 61 | Unemployed | Hypertension, diabetes | Has visited a live poultry market | 7 | 12 | 40.2 | Fever | Nausea, cough, fatigue, chest tightness, headache, apathy | Standard oxygen therapy |
| 14 | Man | 46 | Peasant | Carriers of chronic hepatitis b virus surface antigen | Breeding chickens and ducks | 1 | 1 | 41 | Fever | Nausea, cough, fatigue, chest tightness | Standard oxygen therapy |
| 15 | Man | 40 | Office worker | Hypertension | None | / | 12 | 39.2 | Fever | Nausea, cough, fatigue, muscle pain | Standard oxygen therapy |
| 16 | Woman | 47 | Peasant | None | Breeding chickens and ducks | 1 | 1 | 38 | Fever | Cough, apathy | Standard oxygen therapy |
| 17 | Woman | 64 | Retire | None | Parrot | 1 | 12 | 39.4 | Fever | Cough | Standard oxygen therapy |
| 18 | Woman | 76 | Unemployed | Acute posterior ganglionitis | Has visited a live poultry market | 10 | 12 | 39.4 | Fever | Fever, cough, fatigue, muscle pain | Standard oxygen therapy |
| 19 | Woman | 65 | Retire | None | None | / | 1 | 41.4 | Fever | Nausea, cough, fatigue, chest tightness, chest pain, muscle soreness, headache | Standard oxygen therapy |
| 20 | Man | 74 | Peasant | Chronic obstructive pulmonary | Has visited a live poultry market | 5 | 1 | 39.5 | Cough | Fatigue, chest tightness, apathy | Standard oxygen therapy |
| 21 | Man | 60 | Retire | Malignant tumor of colon | Chicken | 1 | 1 | 39.4 | Fever | Nausea, cough, chest tightness | Standard oxygen therapy |
| 22 | Woman | 61 | Peasant | Nnone | None | / | 2 | 39 | Fever | Nausea, cough, fatigue, muscle pain | Standard oxygen therapy |
| 23 | Woman | 28 | Office worker | None | None | / | 7 | 40.2 | Fever | Nausea, cough, fatigue, muscle pain, chest pain, headache | Standard oxygen therapy |
| 24 | Man | 79 | Peasant | Chronic obstructive pulmonary | None | / | 10 | 39.2 | Fever | Cough, poor appetite, fatigue, chest tightness | Standard oxygen therapy |
| 25 | Man | 69 | Retire | Hypertension, diabetes | Parrot | 14 | 12 | 39.4 | Fever | Nausea, cough, fatigue, chest tightness, muscle soreness | Treatment with high-flow nasal ventilation |
| 26 | Man | 54 | Retire | None | None | / | 12 | 40 | Fever | Nausea, cough, fatigue, muscle soreness, headache | Standard oxygen therapy |
| 27 | Woman | 69 | Retire | None | None | / | 1 | 38.4 | Fever | Nausea, cough, fatigue, chest tightness, muscle soreness | Treatment with high-flow nasal ventilation |
| 28 | Woman | 38 | Unemployed | None | Parrot | 15 | 1 | 40 | Fever | Nausea, cough, fatigue, muscle pain | Standard oxygen therapy |
| 29 | Man | 58 | Unemployed | Diabetes mellitus | None | / | 10 | 40 | Fever | Nausea, cough, fatigue | Standard oxygen therapy |
| 30 | Man | 62 | Retire | None | None | / | 12 | 39.6 | Fever | Nausea, cough, fatigue, headache | Standard oxygen therapy |
| 31 | Man | 82 | Retire | Hypertension, diabetes | Parrot | 1 | 3 | 40.6 | Fever | Nausea, cough, fatigue, chest tightness, muscle soreness, headache | Standard oxygen therapy |
| 32 | Woman | 52 | Retire | None | Pigeon | 1 | 4 | 39.4 | Fever | Nausea, cough, fatigue, muscle soreness, headache | Standard oxygen therapy |
| 33 | Man | 46 | Office worker | None | Parrot | 1 | 4 | 39.5 | Fever | Nausea, cough, fatigue, diarrhea, muscle pain | Standard oxygen therapy |
| 34 | Woman | 75 | Peasant | None | Parrot | 1 | 3 | 40.2 | Fever | Nausea, cough, fatigue, chest pain, headache | Standard oxygen therapy |
