# Supplementary material for: Early diagnosis and therapy of Chlamydia psittaci pneumonia: insights from chest CT, tNGS, and omadacycline in 34 cases
Source: Front Med (Lausanne). 2026 Aug 28;13:1921406. doi: 10.3389/fmed.2026.1921406 (PMC13562005; doi:10.3389/fmed.2026.1921406)
Supplement: Supplementary file 2 [file Table2.docx]

Supplementary table 2 Laboratory findings in 34 patients with *C. psittaci* pneumonia

| **Case** | **WBC**  **（×109/L）** | **N%** | **CRP**  **（mg/L）** | **PCT**  **（ng/mL）** | **LDH**  **（U/L）** | **AST**  **（U/L）** | **ALT**  **（U/L）** | **γ-GGT**  **（U/L）** | **CK**  **（U/L）** | **K（mmol/L）** | **EGFR** | **D- dimer**  **（ng/mL）** |
| --- | --- | --- | --- | --- | --- | --- | --- | --- | --- | --- | --- | --- |
| 1 | 7.2 | 73.5 | 102.6 | 0.45 | 305 | 80 | 58 | 112 | 140 | 4.41 | 107.46 | 242 |
| 2 | 6.7 | 80.3 | 21.2 | 0.07 | 191 | 11 | 19 | 14 | 79 | 4.52 | 86.01 | 111 |
| 3 | 14.2 | 86.4 | 182.8 | 7.12 | 365 | 65 | 31 | 11 | 3400 | 3.18 | 33.69 | 1934 |
| 4 | 10.7 | 85.5 | 96.5 | / | 301 | 39 | 27 | 10 | 262 | 2.83 | 91.01 | 150 |
| 5 | 5.5 | 71.7 | 86 | 0.13 | 322 | 39 | 59 | 31 | 83 | 3.76 | 72.9 | 1955 |
| 6 | 14.2 | 83.9 | 155.3 | 0.58 | 206 | 77 | 97 | 122 | 1434 | 3.41 | 50.2 | 1003 |
| 7 | 5.5 | 58.4 | 37.8 | / | / | 23 | 22 | 19 | / | / | / | / |
| 8 | 4.9 | 82.3 | 226.8 | 0.5 | 456 | 78 | 56 | 17 | 2011 | 3.76 | 120.1 | 3257 |
| 9 | 7.3 | 80.8 | 261.3 | 0.67 | 329 | 50 | 27 | 139 | 591 | 5.08 | 62.5 | 699 |
| 10 | 9.4 | 87.4 | 96.6 | 0.21 | 206 | 52 | 139 | 71 | 37 | 3.68 | 82.8 | 704 |
| 11 | 9.9 | 73.3 | 33.9 | 0.1 | 332 | 143 | 109 | 135 | 46 | 4.18 | 121.4 | 510 |
| 12 | 6 | 91 | 135.1 | 1.48 | 349 | 69 | 123 | 121 | 389 | 4.26 | 70.4 | 2276 |
| 13 | 5.3 | 85.3 | 245.5 | 0.99 | 204 | 13 | 19 | 15 | 217 | 3.65 | 62 | 1408 |
| 14 | 6.8 | 85.1 | 166.7 | 1.43 | 300 | 45 | 36 | 42 | 172 | 4.2 | 104 | 636 |
| 15 | 9.1 | 68.2 | 82.2 | 0.06 | 166 | 19 | 25 | 60 | 82 | 3.83 | 101 | 275 |
| 16 | 9.9 | 84.2 | 130.4 | 0.06 | 234 | 82 | 62 | 19 | 43 | 4.15 | 103.3 | 4672 |
| 17 | 9.4 | 73.9 | 32 | 0.03 | 207 | 26 | 19 | 9 | 97 | 4.11 | 92.4 | 403 |
| 18 | 6.1 | 81.3 | 100.9 | 0.16 | 179 | 19 | 12 | 21 | 138 | 3.3 | 84.8 | 2695 |
| 19 | 13.4 | 81.9 | 189.6 | 0.24 | 222 | 73 | 57 | 35 | 118 | 3.66 | 96 | 1423 |
| 20 | 6 | 87.4 | 261.3 | 2.21 | 423 | 146 | 49 | 95 | 458 | 3.57 | 99.6 | 3889 |
| 21 | 7.9 | 78.4 | 130 | 0.9 | 165 | 22 | 30 | 14 | 53 | 4.57 | 138.9 | 1455 |
| 22 | 6.7 | 88.9 | 286.5 | 0.44 | 271 | 62 | 97 | 245 | 114 | 3.53 | 101.7 | 1480 |
| 23 | 5.8 | 85.7 | 77.6 | 0.19 | 219 | 24 | 17 | 22 | 45 | 3.77 | 125 | 496 |
| 24 | 8.7 | 83 | 56.1 | 0.34 | 316 | 101 | 26 | 21 | 30 | 3.01 | 73.6 | 1968 |
| 25 | 5.1 | 91.1 | 192.3 | 0.14 | 264 | 105 | 155 | 59 | 298 | 4.29 | 72.6 | 274 |
| 26 | 7.5 | 75.8 | 198.7 | 0.08 | 251 | 157 | 126 | 187 | 37 | 3.9 | 120.5 | 879 |
| 27 | 11.2 | 84.6 | 243.7 | 7.22 | 241 | 33 | 11 | 21 | 317 | 4.27 | 47.3 | 952 |
| 28 | 6.1 | 73.3 | 127.2 | 0.48 | 143 | 24 | 13 | 13 | 75 | 4.09 | 80.7 | 523 |
| 29 | 9.1 | 83.5 | 158.3 | 2.57 | 210 | 28 | 83 | 73 | 72 | 3.38 | 92.7 | 572 |
| 30 | 4.3 | 82.3 | 241.8 | 0.13 | 310 | 57 | 69 | 78 | 218 | 3.95 | 90.4 | 1147 |
| 31 | 9.1 | 83.8 | 144.4 | 0.19 | 181 | 22 | 25 | 62 | 130 | 4.32 | 78.6 | 541 |
| 32 | 11.6 | 85.4 | 111.3 | 0.09 | 243 | 113 | 92 | 100 | 138 | 3.68 | 88.9 | 724 |
| 33 | 4.2 | 73.9 | 111.9 | 0.09 | 162 | 55 | 46 | 29 | 35 | 3.65 | 117.5 | 684 |
| 34 | 7.2 | 82.1 | 142.6 | 0.39 | 195 | 45 | 43 | 12 | 116 | 3.64 | 175.2 | 1191 |
