# Supplementary material for: Early diagnosis and therapy of Chlamydia psittaci pneumonia: insights from chest CT, tNGS, and omadacycline in 34 cases
Source: Front Med (Lausanne). 2026 Aug 28;13:1921406. doi: 10.3389/fmed.2026.1921406 (PMC13562005; doi:10.3389/fmed.2026.1921406)
Supplement: Supplementary file 3 [file Table3.docx]

Supplementary table 3 Treatment regimens and prognosis of 34 patients with *C. psittaci* pneumonia

| **Case** | **Antibiotics taken prior to diagnosis** | **Antibiotics after diagnosis** | **Time from onset to hospitalization (days)** | **Time to temperature reduction after administration of effective antibiotics (days)** | **Length of hospital stay**  **(days)** | **final result** | **group** |
| --- | --- | --- | --- | --- | --- | --- | --- |
| 1 | Moxifloxacin + Amoxicillin and Clavulanate Potassium | moxifloxacin | 5 | 3 | 14 | Improvement | B |
| 2 | Moxifloxacin + Amoxicillin and Clavulanate Potassium | moxifloxacin | 4 | 2 | 7 | Improvement | B |
| 3 | Shupushen + Moxifloxacin | moxifloxacin | 3 | 2 | 13 | Improvement | B |
| 4 | piperacillin | Azithromycin | 3 | 2 | 10 | Improvement | B |
| 5 | Levofloxacin | Levofloxacin + Doxycycline | 12 | 2 | 9 | Improvement | B |
| 6 | Meropenem + Levofloxacin | omadacycline | 3 | 1 | 14 | Improvement | A |
| 7 | moxifloxacin | deoxytetracycline | 3 | 2 | 7 | Improvement | B |
| 8 | Piperacillin + Levofloxacin | omadacycline | 4 | 1 | 12 | Improvement | A |
| 9 | Cefoperazone and Sulbactam + Azithromycin | omadacycline | 6 | 1 | 8 | Improvement | A |
| 10 | Cefoperazone and Sulbactam + Levofloxacin | omadacycline | 3 | 1 | 9 | Improvement | A |
| 11 | Levofloxacin | deoxytetracycline | 3 | 4 | 7 | Improvement | B |
| 12 | Piperacillin-tazobactam | omadacycline | 4 | 2 | 8 | Improvement | A |
| 13 | Ceftriaxone, Piperacillin-Tazobactam | omadacycline | 5 | 2 | 14 | Improvement | A |
| 14 | cefatriaxone | omadacycline | 5 | 1 | 7 | Improvement | A |
| 15 | Levofloxacin | omadacycline | 2 | 1 | 6 | Improvement | A |
| 16 | Levofloxacin | Levofloxacin | 7 | 3 | 7 | Improvement | B |
| 17 | Levofloxacin | Levofloxacin + Doxycycline | 1 | 2 | 7 | Improvement | B |
| 18 | Piperacillin-tazobactam + Levofloxacin | omadacycline | 3 | 1 | 11 | Improvement | A |
| 19 | Levofloxacin | Levofloxacin | 6 | 4 | 7 | Improvement | B |
| 20 | Levofloxacin | omadacycline | 3 | 1 | 9 | Improvement | A |
| 21 | Levofloxacin | omadacycline | 3 | 1 | 7 | Improvement | A |
| 22 | Piperacillin-tazobactam + Levofloxacin | omadacycline | 7 | 2 | 10 | Improvement | A |
| 23 | Levofloxacin | omadacycline | 4 | 1 | 7 | Improvement | A |
| 24 | Piperacillin-tazobactam + Levofloxacin | omadacycline | 7 | 2 | 9 | Improvement | A |
| 25 | piperacillin | omadacycline | 5 | 2 | 14 | Improvement | A |
| 26 | Ceftriaxone + Levofloxacin | Levofloxacin | 5 | 5 | 7 | Improvement | B |
| 27 | omadacycline | omadacycline | 3 | 4 | 11 | Improvement | A |
| 28 | Levofloxacin | omadacycline | 5 | 1 | 9 | Improvement | A |
| 29 | moxifloxacin | omadacycline | 5 | 1 | 9 | Improvement | A |
| 30 | Cefalosporin + Levofloxacin | omadacycline | 7 | 1 | 10 | Improvement | A |
| 31 | Levofloxacin | omadacycline | 3 | 1 | 10 | Improvement | A |
| 32 | Levofloxacin | Levofloxacin | 5 | 3 | 10 | Improvement | B |
| 33 | Levofloxacin | Levofloxacin | 3 | 3 | 6 | Improvement | B |
| 34 | Piperacillin-tazobactam | omadacycline | 7 | 1 | 12 | Improvement | A |
